# Catabolic Division of Labor Enhances Production of D-Lactate and Succinate From Glucose-Xylose Mixtures in Engineered *Escherichia coli* Co-culture Systems

**DOI:** 10.3389/fbioe.2020.00329

**Published:** 2020-05-05

**Authors:** Andrew D. Flores, Hyun G. Choi, Rodrigo Martinez, Moses Onyeabor, E. Zeynep Ayla, Amanda Godar, Michael Machas, David R. Nielsen, Xuan Wang

**Affiliations:** ^1^Chemical Engineering, School for Engineering of Matter, Transport, and Energy, Arizona State University, Tempe, AZ, United States; ^2^School of Life Sciences, Arizona State University, Tempe, AZ, United States

**Keywords:** division of labor, co-culture, biomass conversion, lactate, succinate

## Abstract

Although biological upgrading of lignocellulosic sugars represents a promising and sustainable route to bioplastics, diverse and variable feedstock compositions (e.g., glucose from the cellulose fraction and xylose from the hemicellulose fraction) present several complex challenges. Specifically, sugar mixtures are often incompletely metabolized due to carbon catabolite repression while composition variability further complicates the optimization of co-utilization rates. Benefiting from several unique features including division of labor, increased metabolic diversity, and modularity, synthetic microbial communities represent a promising platform with the potential to address persistent bioconversion challenges. In this work, two unique and catabolically orthogonal *Escherichia coli* co-cultures systems were developed and used to enhance the production of D-lactate and succinate (two bioplastic monomers) from glucose–xylose mixtures (100 g L^–1^ total sugars, 2:1 by mass). In both cases, glucose specialist strains were engineered by deleting *xylR* (encoding the xylose-specific transcriptional activator, XylR) to disable xylose catabolism, whereas xylose specialist strains were engineered by deleting several key components involved with glucose transport and phosphorylation systems (i.e., *ptsI*, *ptsG*, *galP*, *glk*) while also increasing xylose utilization by introducing specific *xylR* mutations. Optimization of initial population ratios between complementary sugar specialists proved a key design variable for each pair of strains. In both cases, ∼91% utilization of total sugars was achieved in mineral salt media by simple batch fermentation. High product titer (88 g L^–1^ D-lactate, 84 g L^–1^ succinate) and maximum productivity (2.5 g L^–1^ h^–1^ D-lactate, 1.3 g L^–1^ h^–1^ succinate) and product yield (0.97 g g-total sugar^–1^ for D-lactate, 0.95 g g-total sugar^–1^ for succinate) were also achieved.

## Introduction

Production of D-lactate (LA) and succinate (SA) from renewable carbohydrate feedstocks provides a sustainable and greener alternative to their petroleum-based production (Abdel-Rahman et al., 2013; Ahn et al., 2016; Es et al., 2018). LA and SA serve as two important monomers in the production of biodegradable plastics, including poly(butylene succinate) (PBS) and poly(lactic acid) (PLA), respectively. SA is largely produced via petroleum-derived maleic anhydride, and only a handful of plants producing bio-based SA currently exists (Jansen and van Gulik, 2014). Meanwhile, ∼95% of global LA production occurs via fermentation, being derived almost entirely from costly raw materials such as grain starch or sucrose from sugar cane, feedstocks that compete with the food chain (Okano et al., 2010; Abdel-Rahman et al., 2011). Alternatively, lignocellulose-derived sugars from non-food carbohydrates such as agricultural residues, forest products, or energy crops represent an attractive feedstock for producing bio-based plastics due to their increased abundance and sustainability, as well as lower cost (Lynd, 2017). The two most abundant sugars in most lignocellulosic biomass is glucose (a hexose, accounting for ∼30–50% dry weight) from the cellulose fraction and xylose (a pentose, constituting ∼20–35% dry weight) from the hemicellulose fraction (Nieves et al., 2015). Minute quantities of other fermentable sugars (i.e., arabinose, galactose, mannose) are additionally found in lignocellulosic biomass (Nieves et al., 2015).

**FIGURE 1:**
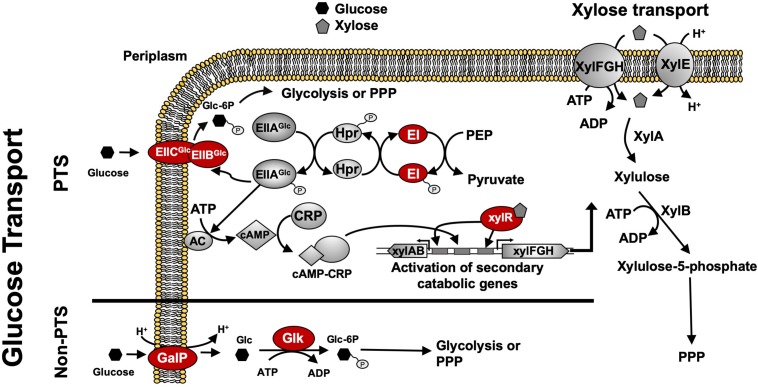
Regulatory and catabolic pathways for glucose and xylose metabolism in *E*. *coli*. The primary mechanism for glucose transport in *E*. *coli* is through the phosphoenolpyruvate: sugar phosphotransferase system (PTS), including components EIIBC^Glc^, EIIA^Glc^, Hpr, and EI (encoded by *ptsG*, *crr*, *ptsH*, and *ptsI*, respectively). Under high glucose concentrations, glucose is transported via EIICB^Glc^, leading to more abundant unphosphorylated EIICA^Glc^ that inhibits adenylate cyclase (AC) and lowers cAMP levels. Glucose-induced catabolite repression is mainly caused by low levels of cAMP which leads to non-functional CRP. Without CRP activation, the transcriptional activation of xylose catabolism pathways cannot be achieved. Under low glucose concentrations, AC is active due to the decreased amount of unphosphorylated EIICA^Glc^, thus leading to increased cAMP levels and activated CRP. Both activated CRP and XylR (activated when bound by xylose) are required to activate the xylose catabolic operons, *xylAB* and *xylFGH*. Phosphorylated sugar intermediates enter glycolysis or PPP pathways for full degradation. GalP functions as an alternative non-PTS glucose transporter and *glk* encodes cytoplasmic glucokinase to phosphorylate glucose for further glycolysis. Glucose uptake systems including both PTS components and GalP as well as Glk and XylR indicated in red ovals are genetic engineering targets to construct sugar specialists. Abbreviation: AC, adenylate cyclase; CRP, cAMP receptor protein; PPP, pentose phosphate pathway; Glc, glucose; Glc-6P, glucose 6-phosphate; Xyl; xylose.

For many native and engineered bacteria, the inability to efficiently co-utilize sugar mixtures in mineral salts medium at high catabolic rates (e.g., > 2 g L^–1^h^–1^ for each sugar) is due to a complex, global regulatory phenomenon known as carbon catabolite repression (CCR), which often results in incomplete and/or sequential sugar utilization. For instance, in *Escherichia coli*, this sequential sugar preference is controlled via the coordinated action of the global transcriptional regulator cyclic AMP (cAMP) receptor protein (CRP) along with a second regulator specific to the secondary sugars of interest, such as xylose. Activation of the requisite xylose catabolism operons (i.e., *xylFGH* and *xylAB*) requires both activated CRP (active when bound by cAMP) and XylR (regulator specific for xylose catabolism, active when bound by xylose) (Song and Park, 1997; Figure 1). When wild-type *E. coli* ferments glucose–xylose mixtures, for example, cAMP levels are low because abundant extracellular glucose leads to the active mode of the phosphotransferase system (PTS), increasing the abundance of unphosphorylated PTS components (IIA protein) and inhibiting the activity of adenylyl cyclase (AC; catalyzing cAMP synthesis). Xylose catabolism thus does not occur due to the lack of activated CRP and, as a result, initiates only after glucose is mostly utilized and phosphorylated IIA protein activates AC, leading to high cAMP levels (Figure 1).

To date, several engineered microorganisms producing LA [e.g., lactic-acid bacteria (LAB), *Saccharomyces cerevisiae, E. coli*] and SA (e.g., *Mannheimia succiniciproducens, Corynebacterium glutamicum, Bacillus* strains) have been reported using various substrates (Grabar et al., 2006; Ishida et al., 2006; Wang et al., 2011; Litsanov et al., 2012; Awasthi et al., 2018). While different strains have their own unique advantages/disadvantages (e.g., ease of genetic manipulation, product tolerance, and other physiological benefits), from a bioprocessing perspective, it is desirable that it should also be capable of rapidly and simultaneously utilizing the substrate at high initial loadings (e.g., ≥ 100 g L^–1^ total sugar). Under such conditions, *E*. *coli* has proven to be a particularly promising biocatalyst for the production of both LA and SA. In particular, via a combination of engineering strategies, *E. coli* strains have been developed to produce both LA and SA at high yields (>90%) and titers (>90 g L^–1^) and maximum productivities (>1.0 g L^–1^ h^–1^) (Sawisit et al., 2015; Utrilla et al., 2016; Sievert et al., 2017). Despite these achievements, however, challenges still remain with respect to the efficient conversion of glucose–xylose mixtures.

Owing to unique features such as strain-specific specialization and metabolic modularity, the engineering and use of synthetic microbial communities represent a promising bioprocessing strategy (Zhang et al., 2015; Zhou et al., 2015; Camacho-Zaragoza et al., 2016; Jones et al., 2017), with the potential to surmount many limitations faced by traditional monocultures (Lu et al., 2019; Roell et al., 2019). Through catabolic division of labor, for example, engineered co-cultures have specifically emerged as an effective strategy for achieving efficient co-utilization of different mixtures of lignocellulose-derived sugars (Eiteman et al., 2008; Zhang et al., 2015; Chappell and Nair, 2017; Wang et al., 2019). Eiteman et al. (2008) first demonstrated the utility of this approach, engineering a co-culture composed of *E*. *coli* sugar specialist strains to co-utilize glucose–xylose mixtures (∼14 g L^–1^ total sugars). This general strategy was later expanded upon by others to develop a three-member community of *E*. *coli* specialists to co-utilize a mixture of glucose, galactose, and mannose (∼7.5 g L^–1^ total sugars) (Chappell and Nair, 2017). Most recently, meanwhile, our group engineered two different catabolically orthogonal co-culture systems (derived from wild-type *E*. *coli* W or ethanologenic *E*. *coli* LY180), each capable of co-utilizing 100 g L^–1^ of a glucose–xylose mixture (2:1 by weight) in mineral salt media by simple batch fermentation (Flores et al., 2019). In this work, we further explore the utility of this strategy by applying analogous principles to engineer two unique co-culture systems composed of catabolically orthogonal *E*. *coli* strains for the production of LA and SA from glucose–xylose mixtures. In both cases, optimization of initial population ratios between each strain pair proved a key design variable toward achieving efficient conversion of the feedstock mixture along with high production metrics. This strategy of “population-level” tuning helps to alleviate biosynthetic burden while circumventing technical difficulties that would otherwise accompany the more traditional optimization of multiple catabolic pathways in a single strain.

## Results and Disccusion

### Construction of Catabolically Orthogonal Sugar Specialists for D-Lactate Production

TG114 (a derivative of *E*. *coli* KO11 and based on *E*. *coli* W) has previously been shown to produce LA at maximum volumetric productivity (2.88 g L^–1^ h^–1^), titer (118 g L^–1^), yield (98%), and chiral purity (>99.9%) in mineral salts medium containing 120 g L^–1^ glucose (Grabar et al., 2006). In spite of this, however, poor performance is apparent in sugar mixtures as a result of CCR (Supplementary Figures S1A–D). Specifically, when cultured using a 100 g L^–1^ glucose–xylose mixture (2:1 by mass), TG114 utilized 88% of the provided glucose within the first 24 h and 100% by 48 h, but only 38% of the supplied xylose by 96 h, corresponding to only ∼80% total sugar utilization. LA was produced, meanwhile, at overall and maximum volumetric productivities (*Q*_*LA*_) of 0.80 ± 0.01 and 2.6 ± 0.4 g L^–1^ h^–1^, a final titer of 77 ± 1 g L^–1^, and yield (*Y*_*p/s*_) of 0.96 ± 0.02 g g-total sugars^–1^ (note: all reported yields based on total sugars consumed). To overcome the sugar co-utilization bottleneck experienced by TG114, a division of labor approach was used to construct a pair of complementary, catabolically orthogonal specialist strains, each capable of catabolizing either glucose or xylose but not both sugars (Supplementary Figure S2A). Specifically, a glucose specialist strain, TGglc, was constructed by deleting the xylose-specific transcriptional activator XylR (encoded by *xylR*) to inactivate xylose catabolism. A xylose specialist strain, TGxyl, was constructed by deleting the major components of glucose transport and its initial catabolism (i.e., *ptsI*, *ptsG*, *galP, glk*) (Figure 1 and Table 1). To further enhance xylose utilization of this specialist strain, wild-type XylR was also replaced with a mutant copy [P363S and R121C; denoted as XylR^∗^ and reported to enable a stronger activation of the D-xylose catabolic genes (Sievert et al., 2017)].

**TABLE 1:** List of strains and plasmids used in this study.

Strains and plasmids	Relevant characteristics	Source
**Strains**		
TG114	ATCC 9637 Δ*pflB frdBC*:FRT *adhE*:FRT *ackA*:FRT *mgsA*:FRT evolved for converting glucose to D-lactate	Grabar et al., 2006
TGglc	TG114 Δ*xylR*	This study
TGxyl	TG114 Δ*ptsI*Δ*ptsG*Δ*galP glk*:*kan*^R^ (Kan^R^) *xylR:xylR^∗^*	This study
KJ122	ATCC 8739 pck^*A^ ptsI^*B^ Δ*ldhA* Δ*adhE* Δ*ackA*, Δ(*focA-pflB*) Δ*mgsA* Δ*poxB* Δ*tdcDE* Δ*citF* Δ*aspC* Δ*sfcA*	Jantama et al., 2008
KJglc	KJ122 *xylR*:*tetA-sacB* (Tet^R^)	This study
KJxyl	KJ122 Δ*galP*Δ*ptsI glk*:*kan*^R^ (Kan^R^) *xylR:xylR^∗^* quickly adapted in glucose-xylose mixture	This study
T-SACK	W3110 *ara*D < > *tetA-sacB-amp flic* < > *cat argG:Tn*5	Li et al., 2013
**Plasmids**		
pXW001	The *cat-sacB* cassette with the *sacB* native terminator cloned into a modified vector pLOI4162	Sievert et al., 2017
pKD46	Red recombinase, temperature-conditional, *bla*	Datsenko and Wanner, 2000

*^*A*^pck^∗^ denotes a mutated form of pck (G to A at position -64 relative to the ATG start codon). ^*B*^ptsI^∗^ denotes a mutated form of ptsI (single-base deletion at position 1,673 causing a frameshift mutation in the carboxyl-terminal region.*

**FIGURE 2:**
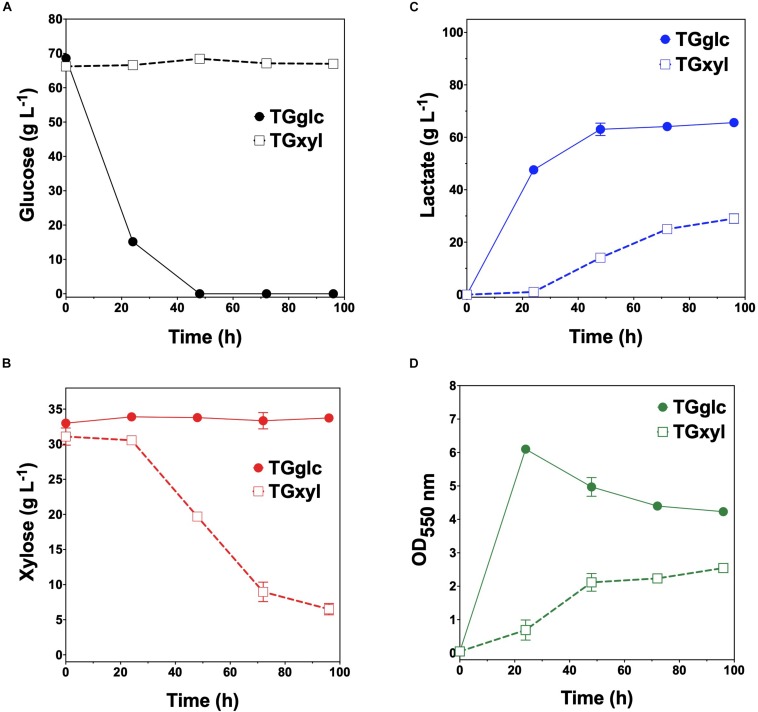
Fermentation of glucose–xylose mixtures (2:1 by mass) by lactate-producing sugar specialist strains. Concentrations of **(A)** glucose, **(B)** xylose, **(C)** D-lactate, and **(D)** OD_550nm_ measured in the fermentation broth for TGglc (closed circles) and TGxyl (open squares). All fermentations were performed in batch mode and in mineral salts media. Data points represent the arithmetic mean of three replicates, and the error bars represent one standard deviation.

Consistent with their respective genotypes, TGglc and TGxyl each preferentially utilized only one sugar when fermented in mineral salt media supplemented with 66 g L^–1^ glucose and 33 g L^–1^ xylose (Figures 2A,B and Table 2). TGglc utilized 100% of the supplied glucose within 48 h (77% within the first 24 h, similar to TG114) and virtually no xylose. This resulted in a maximum *Q*_*LA*_ of 2.0 ± 0.1 g L^–1^ h^–1^ (overall, *Q*_*LA*_ was 0.68 ± 0.01 g L^–1^ h^–1^), final LA titer of 66 ± 1 g L^–1^, and *Y*_*p/s*_ of 0.96 g g-total sugars ^–1^ (Figures 2C,D and Table 2). In contrast, under the same conditions, TGxyl consumed just ∼80% of supplied xylose by 96 h and no glucose (Figures 2A,B). Growth of TGxyl, meanwhile, was significantly less than that of TGglc and TG114 [1.1 ± 0.1 g-dry cell weight (DCW) L^–1^ compared to 2.7 ± 0.1 and 2.9 ± 0.2 gDCW L^–1^, respectively]. While this difference is at least in part due to the lower energy yield of xylose relative to glucose, it is also possible that, since TG114 was originally engineered for and adapted in mineral salt media containing only glucose as carbon source (Grabar et al., 2006), it may have only gained mutations specifically tailored for glucose catabolism. Despite its slower growth rate and reduced biomass accumulation, TGxyl still produced LA at a final titer of 25 ± 1 g L^–1^, *Y*_*p/s*_ of ∼0.99 g g-total sugars ^–1^, and maximum *Q*_*LA*_ of 0.50 ± 0.08 g L^–1^ h^–1^ (Figures 2C,D and Table 2).

**TABLE 2:** Comparing the performance of individual *E. coli* sugar specialists and co-cultures during D-lactate (LA) and succinate (SA) fermentation.

Strains(s)	Sugar utilized (%)			Biomass^B^	*Q* (g L^–1^ h^–1^)^C^			*Y*_*p/s*_	Titer
			
Fermented	Glucose	Xylose	Total^A^	gDCW L^–1^	Glucose	Xylose	Product	g g^–1D^	g L^–1^
**Lactate**									
TGglc	100 ± 0	NC	68 ± 1	2.7 ± 0.1	2.2 ± 0.1	NR	2.0 ± 0.1	0.96 ± 0.01	65 ± 1
TGxyl	NC	80 ± 2	25 ± 1	1.1 ± 0.1	NR	0.44 ± 0.01	0.50 ± 0.08	0.99 ± 0.1	25 ± 1
TGglc:TGxyl Ratio 1:1	100 ± 0	22 ± 1	75 ± 1	2.8 ± 0.1	5.5 ± 0.4	0.35 ± 0.04	5.8 ± 0.5	0.99 ± 0.04	73 ± 2
TGglc:TGxyl Ratio 1:50	100 ± 0	58 ± 2	86 ± 1	2.7 ± 0.1	3.2 ± 0.6	0.44 ± 0.04	3.7 ± 1.1	0.95 ± 0.02	84 ± 2
TGglc:TGxyl Ratio 1:100	100 ± 0	71 ± 3	91 ± 1	2.7 ± 0.3	2.4 ± 0.2	0.52 ± 0.05	2.5 ± 0.2	0.97 ± 0.01	88 ± 1
**Succinate**									
KJglc	100 ± 0	NC	71 ± 1	2.9 ± 0.1	2.1 ± 0.1	NR	1.9 ± 0.1	0.88 ± 0.01	65 ± 1
KJxyl	NC	87 ± 2	28 ± 2	2.7 ± 0.2	NR	0.60 ± 0.06	0.69 ± 0.20	1.21 ± 0.05	33 ± 1
KJglc:KJxyl Ratio 1:1	93 ± 5	89 ± 2	91 ± 4	3.1 ± 0.2	0.84 ± 0.1	0.72 ± 0.04	1.3 ± 0.1	0.95 ± 0.01	84 ± 1
KJglc:KJxyl Ratio 1:50	19 ± 14	86 ± 2	39 ± 9	2.8 ± 0.2	0.49 ± 0.2	0.73 ± 0.1	0.82 ± 0.1	0.97 ± 0.1	37 ± 4
KJglc:KJxyl Ratio 1:100	6 ± 4	88 ± 1	31 ± 3	2.9 ± 0.1	0.2 ± 0.1	0.66 ± 0.06	0.69 ± 0.02	0.94 ± 0.1	29 ± 1
KJglc:KJxyl Ratio 2:1	100 ± 0	46 ± 4	83 ± 1	3.4 ± 0.1	1.4 ± 0.1	0.30 ± 0.09	2.1 ± 0.1	0.84 ± 0.08	76 ± 6

*All cultures were initially supplied with ∼100 g L^–1^ of glucose–xylose mixtures (ratio 2:1 by mass). No Consumption (NC) < 1% sugar utilized; No Rate (NR) < 0.1 g L^–1^ h^–1^. ^*A*^Total sugar utilized per sugar supplied. ^*B*^Dry cell weight (DCW) values are calculated from maximum OD_550nm_ (0.44 gDCW L^–1^ with an optical density of 1.0 at 550 nm). ^*C*^Maximum volumetric rates (Q) are calculated when the slope of substrate utilization or product formation is most linear. ^*D*^Y_*p/s*_ denotes the product yield coefficient and is calculated as gram product per gram total sugar utilized.*

### Engineering and Optimizing a Synthetic Co-culture for Efficient Conversion of Glucose–Xylose Mixtures to D-Lactate

**FIGURE 3:**
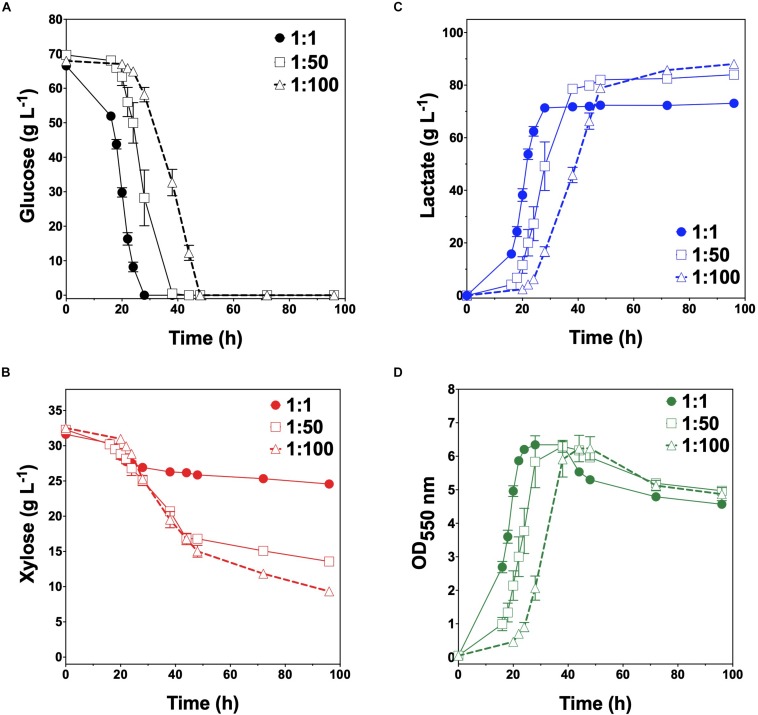
Fermentation of glucose–xylose mixtures (2:1 by mass) co-cultures composed of catabolically orthogonal lactate-producing specialist strains. Concentrations of **(A)** glucose, **(B)** xylose, **(C)** D-lactate, and **(D)** OD_550nm_ measured in the fermentation broth for TGglc:TGxyl co-cultures using an initial inoculum ratio of 1:1 (closed circles), 1:50 (open squares) and 1:100 (open triangles). All fermentations were performed in batch mode and in mineral salts media. Data points represent the arithmetic mean of three replicates, and the error bars represent one standard deviation.

Given their promising performance metrics with respect to LA production and minimal cross-catabolic activities, TGglc and TGxyl were next used as complementary specialist strains with which to engineer a synthetic co-culture. To balance catabolic rates, simple titration of the initial inoculum ratio between TGglc and TGxyl (e.g., 1:1, 1:50, 1:100) while maintaining a constant total initial OD_550nm_ of 0.05 (the same initial OD_550nm_ as in monoculture fermentations) was performed. As shown in Figure 3A and Table 2, glucose was completely utilized within 48 h for all ratios (similar to TG144 and TGglc monocultures). However, as a result of tuning the initial population, initial volumetric rates of glucose utilization (*Q_*G*__*l*__*c*_*) were subsequently reduced over the first 24 h in a manner proportional to the relative abundance of TGglc (2.4 ± 0.1, 0.82 ± 0.40, and 0.13 ± 0.02 g L^–1^ h^–1^ for ratios 1:1, 1:50, and 1:100, respectively), along with initial rates of biomass accumulation (Figure 3D and Table 2). The corresponding profiles of xylose fermentation (Figure 3B), meanwhile, revealed the opposite and expected effect with respect to xylose catabolism; increasing abundance of TGxyl in the initial inoculum improves xylose utilization. More specifically, at equal abundance (i.e., 1:1), total xylose utilization reached merely 22% by 96 h. This corresponded to 5% less total sugar utilization than by TG114 monocultures (75 vs. 80% total sugar utilization, respectively; Table 2). However, by tuning the initial inoculum ratio to 1:50, 2.6-, and 1.5-fold increases in xylose utilization (58% total xylose used) were realized relative to co-cultures with a 1:1 initial inoculum ratio and TG114 monocultures, respectively (Table 2). Owing to this significant increase in xylose co-utilization, final LA titers achieved by 1:50 co-cultures reached 84 ± 2 g L^–1^ (∼11% higher than the 1:1 co-culture; Figure 3C), while still maintaining high overall performance metrics (*Y*_*p/s*_ of 0.96 g g-total sugars^–1^, maximum *Q*_*LA*_ of 3.7 ± 1.1 g L^–1^ h^–1^; Table 2). Further increasing the relative initial abundance of TGxyl, 1:100 co-cultures then enabled further increased utilization of supplied xylose, in this case reaching ∼71% (Figure 3B). Moreover, total sugar utilized reached 91% by the end of the 96 h fermentation, achieving a final LA titer of 88 ± 1 g L^–1^, *Y*_*p/s*_ of ∼0.97 g g-total sugars^–1^, and maximum *Q*_*LA*_ of 2.5 ± 0.2 g L^–1^ h^–1^ (Table 2). Based on the promising trends observed with 1:50 and 1:100 co-cultures, additional tuning of the inoculum ratio to further increase the initial abundance of TGxyl was subsequently performed, in this case at both 1:500 and 1:1,000 (data not shown). However, no further performance enhancements were realized in terms of either total sugar utilization or LA production in such co-cultures, suggesting that the optimal initial inoculum ratio for TGglc:TGxyl exists at least close to 1:100. Thus, further improvement of this co-culture should next focus on enhancing the inherent properties of each individual strain; for example, increasing rates of xylose catabolism in sugar mixtures by TGxyl through adaptation and/or genetic engineering.

LA production performance demonstrated by the 1:100 co-culture compares well to that of other co-cultures previously engineered for the same purpose, as well as those developed to produce other related fermentation products (Table 3). In particular, Eiteman et al. (2009) developed an *E*. *coli* co-culture composed of glucose and xylose specialists capable of co-utilizing and converting a sugar mixture (∼47 g L^–1^ total sugar, ratio of glucose to xylose is 1.5:1 by mass) to LA (final titer of 32 g L^–1^, *Y*_*p/s*_ of 0.68 g g-total sugars^–1^) in a two-stage, aerobic–anaerobic process (Table 3). In this case, rather than tuning the initial population ratio, a sequential inoculation strategy was instead employed to balance the contribution of each specialist to the net catabolic activity, allowing more time initially for the xylose specialist strain to accumulate under aerobic conditions. Upon reaching the anaerobic phase, the population ratio in their co-culture was estimated as 2:3 glucose:xylose specialists, which similarly illustrates a need for increased abundance of the xylose specialist in this fermentation. In comparison to LA-producing monocultures, Sievert et al. (2017) demonstrated that substituting wild-type *xylR* with *xylR*^∗^ (R121C and P363S; the same mutations used to develop TGxyl in this study) in TG114 enabled co-utilization of 50 g L^–1^ glucose and 43 g L^–1^ xylose (from 100 g L^–1^ glucose–xylose mixture, initially 1:1 by mass) to 86 g L^–1^ LA in mineral salt medium. While minor improvement in terms of sugar utilization was achieved using the present co-culture system, a unique advantage of this approach is the facile tunability that it provides. In this case, catabolic rates can be titrated to achieve optimal fermentation performance by altering initial inoculum ratios between the two specialists. This ability will likely be beneficial when utilizing feedstocks of varying compositions and can be extended beyond simply binary sugar mixtures.

**TABLE 3:** Comparing the performance of different *E*. *coli* co-cultures engineered to convert glucose and xylose to fermentative products.

Media and fermentation condition(s)	Base strain and key mutations^A^	Product(s)	Performance metric(s)^B^	References
**Modified AM1** 6.6% Glucose 3.4% Xylose	**Glucose**: TG114 (an *E. coli* W derivative engineered for lactate production) Δ*xylR*	D-Lactate	*Q*_*Glc*_ ≈ 2.4 g L^–1^ h^–1^ *q*_*Glc*_ ≈ 886 mg gDCW^–1^ h^–1^ *Q*_*Xyl*_ ≈ 0.5 g L^–1^ h^–1^ *q*_*Xy*__*l*_ ≈ 223 mg gDCW^–1^ h^–1^ Total Sugar Utilized ≈ 91g L^–1^ Titer ≈ 88g L^–1^ Productivity ≈ 2.5 g L^–1^ h^–1^ *Y*_*p/*__*s*_ ≈ 0.97	This study
**Batch** Microaerobic	**Xylose:** TG114 Δ*ptsI* Δ*ptsG* Δ*galP* Δ*glk xylR*:*xylR*^∗^			
**Basal** 3.1% Glucose 2.0% Xylose	**Glucose**: *E. coli* MG1655 *xylA748*:FRT *pflB*:Cam	D-Lactate	*q*_*Glc*_ ≈ 540 mg gDCW^–1^ h^–1^ *q*_*Xyl*_ ≈ 325 mg gDCW^–1^ h^–1^ Total Sugar Utilized ≈ 47 g L^–1^ Titer ≈ 32 g L^–1^ *Y*_*p/s*_ ≈ 0.68	Eiteman et al., 2009
**Batch** Aerobic–anaerobic^C^	**Xylose:** *E. coli* MG1655 *pflB*:Cam *ptsG763*:FRT *manZ743*:FRT *glk-726*:FRT			
**Modified AM1** 6.6% Glucose 3.4% Xylose	**Glucose:** KJ122 (an *E*. *coli* C derivative engineered for succinate production) *xylR*:*tetA-sacB*	Succinate	*Q*_*Glc*_ ≈ 0.84 g L^–1^ h^–1^ *q*_*Glc*_ ≈ 188 mg	This study
**Batch** Microaerobic	**Xylose:** KJ122 Δ*galP* Δ*ptsI glk:Kan^R^ xylR:xylR*^∗^		gDCW^–1^ h^–1^ *Q*_*Xyl*_ ≈ 0.72 g L^–1^ h^–1^ *q*_*Xyl*_ ≈ 276 mg gDCW^–1^ h^–1^ Total Sugar Utilized ≈ 91 g L^–1^ Titer ≈ 84 g L^–1^ Productivity ≈ 1.3 g L^–1^ h^–1^ *Y*_*p/s*_ ≈ 0.95 g g^–1^	
**Basal Initial Sugar:** 3% Glucose 1% Xylose	**Glucose:** *E. coli* ATCC8739 *ptsG*:FRT *xylA*:FRT *pflB*:FRT *ldhA*:Kan^R^	Succinate	Titer ≈ 45 g L^–1^ Productivity ≈ 1.7 g L^–1^ h^–1^ *Y*_*p/*__*s*_ ≈ 0.97 g g^–1^	Xia et al., 2015
**Fed:** 1.5% Glucose 0.5% Xylose Fed-Batch Aerobic–Anaerobic^D^	**Xylose:** *E. coli* ATCC8739 *ptsG*:FRT *glk*:FRT *manZ*:FRT *crr*:FRT *ldhA*:FRT *pflB*:FRT *ppc*:Kan^R^			
**Basal** 1.5% Glucose 1.5% Xylose 0.2% Acetate Batch and Fed-Batch Aerobic	**Glucose:** *E. coli* C *xylA748*:FRT *ace732*:FRT *ldhA744*:FRT *poxB772*:FRT *pps-776*: Kan^R^	Pyruvate	**Batch:** Titer ≈ 19 g L^–1^ Yield ≈ 61% Productivity ≈ 1.44 g L^–1^ h^–1^ **Fed-Batch:** Titer ≈ 39 g L^–1^ Productivity ≈ 1.65 g L^–1^ h^–1^	Maleki et al., 2018
	**Xylose:** *E. coli* C *ptsG763*:FRT *glk-726*:FRT *manZ743*:FRT *aceE732*:FRT *ldhA744*:FRT *poxB772*:FRT *pps-776*: Kan^R^			
**Modified AM1** 6.6% Glucose 3.4% Xylose	**Glucose:** LY180 (an *E. coli* W derivative engineered for ethanol production) Δ*xylR* adapted in glucose-xylose	Ethanol	*q*_*Glc–Max*_ ≈ 620 mg DCW^–1^ h^–1^ *q*_*Xyl–Max*_ ≈ 300 mg DCW–1 h^–1^ Total Sugar Utilized ≈ 98 g L^–1^ Titer ≈ 46 g L^–1^ Productivity ≈ 488 mg L^–1^ h^–1^ *Y*_*p/s*_ ≈ 0.45 g g^–1^	(Flores et al., 2019)
**Batch** Microaerobic	**Xylose:** LY180 Δ*ptsI* Δ*ptsG* Δ*galP glk*:Kan^R^ *xylR*:*xylR*^∗^			

*^*A*^Gene deletion, disruption, or modification. ^*B*^q_*Glc*_, specific rate of glucose utilization; Q_*Glc*_, volumetric rate of glucose utilization; q_*Xyl*_, specific rate of xylose utilization; Q_*Xyl*_, volumetric rate of xylose utilization (each calculated when the rate of utilization was approximately constant); Dry cell weight (DCW) calculated from maximum OD_550nm_ (0.44 gDCW L^–1^ with an optical density of 1.0 at 550 nm). ^*C*^Fermentation consisted of two stages: initial aerobic growth followed by an anaerobic phase. ^*D*^Minor amounts of succinate, acetate, and ethanol were also detected.*

Finally, one intriguing observation associated with the developed LA co-culture system was that the volumetric rate of xylose utilization (*Q_*X*__*yl*_*) was found to consistently and abruptly decrease in all co-cultures upon exhaustion of available glucose. For instance, as seen in Figures 3A,B, prior to glucose exhaustion, maximum *Q_*X*__*yl*_* values were ∼0.35 ± 0.04, 0.44 ± 0.04, and 0.52 ± 0.05 g L^–1^ h^–1^ for 1:1, 1:50, and 1:100 co-cultures, respectively. However, following glucose exhaustion, *Q_*X*__*yl*_* in the same co-cultures then dropped to just 0.034 ± 0.009, 0.062 ± 0.004, and 0.12 ± 0.02 g L^–1^ h^–1^. It is unlikely that LA or by-product toxicity is responsible for this behavior since the parent strain (TG114) has been shown to achieve LA titers up to 120 g L^–1^, and almost no other side products are detected during its fermentation (Grabar et al., 2006). This observation possibly suggests that, although the two strains were engineered to be catabolically orthogonal, interstrain interactions certainly do occur throughout these synthetic co-cultures. The exact nature and extent of this behavior remain unknown, however, and warrant further investigation.

### Construction of Catabolically Orthogonal Sugar Specialists for Succinic Acid Production

**FIGURE 4:**
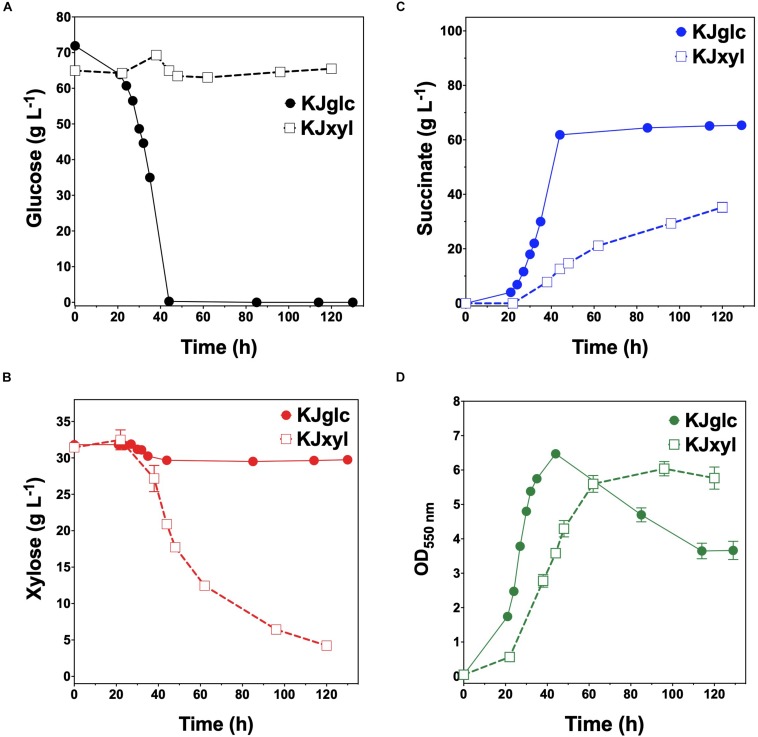
Fermentation of glucose–xylose mixtures (2:1 by mass) by succinogenic sugar specialist strains. Concentrations of **(A)** glucose, **(B)** xylose, **(C)** succinate, and **(D)** OD_550nm_ measured in the fermentation broth for KJglc (closed circles) and KJxyl (open squares). All fermentations were performed in batch mode and in mineral salts media. Data points represent the arithmetic mean of three replicates, and the error bars represent one standard deviation.

To further investigate the generalizable nature of this co-culture strategy along with sets of specific genetic modifications used to create each sugar specialist, the same methodologies were next analogously applied to SA production from glucose–xylose mixtures. In this case, the succinogenic strain KJ122 (a derivative of *E*. *coli* ATCC 8739) was used as the common parent for constructing the two sugar specialist strains: KJglc and KJxyl (Table 1 and Supplementary Figure S2). KJ122 was previously engineered and shown to ferment 100 g L^–1^ glucose to SA (final titer of 82 g L^–1^, overall *Q*_*SA*_ of 0.88 g L^–1^ h^–1^, *Y*_*p/*__*s*_ of 0.90 g g-total sugars^–1^) in mineral salts media (Jantama et al., 2008). Similar to TGglc, batch fermentation of KJglc also revealed virtually no xylose utilization (Figure 4B). In this case, glucose was completely utilized within 42 h at a maximum *Q_*G*__*l*__*c*_* of 2.1 ± 0.1 g L^–1^ h^–1^, while SA was produced at a maximum *Q*_*SA*_ of 1.9 ± 0.1 g L^–1^ h^–1^. At this output, the performance of KJglc was similar to that of its parent strain, KJ122 (Figure 4A, Table 2, and Supplementary Figures S1A–D). Likewise, and as expected, KJxyl was unable to utilize glucose throughout the 120 h fermentation (Figure 4A), but utilized 87% of supplied xylose, leaving just 5 g L^–1^ unused (Figure 4B and Table 2). With maximum *Q*_*Xyl*_ and *Q*_*SA*_ of 0.60 ± 0.06 and 0.69 ± 0.20 g L^–1^ h^–1^, respectively, overall performance of KJxyl was also similar to that of KJ122 (Supplementary Figure S1A).

### Engineering and Optimizing a Synthetic Co-culture for Efficient Conversion of Glucose– Xylose Mixtures to Succinic Acid

**FIGURE 5:**
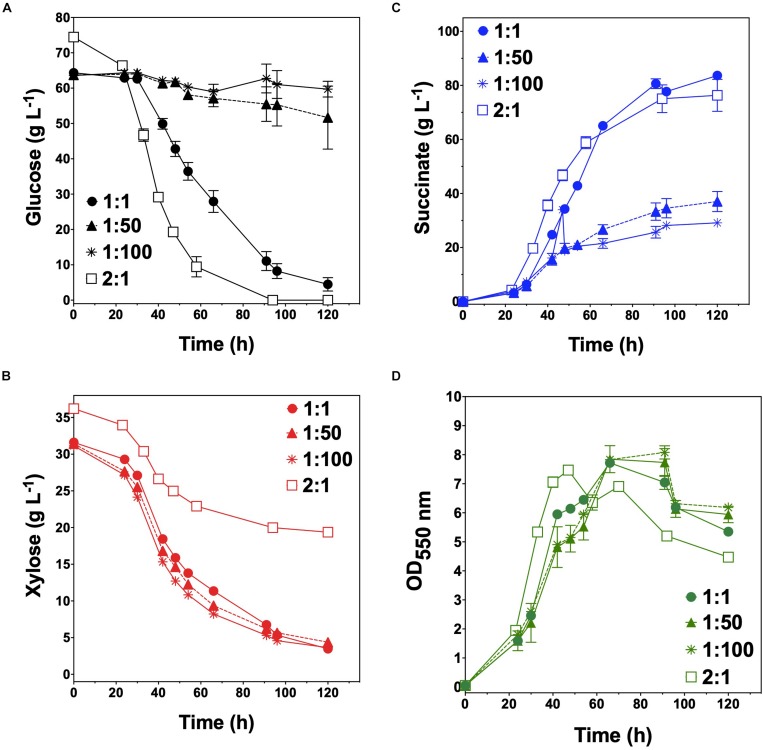
Fermentation of glucose–xylose mixtures (2:1 by mass) co-cultures composed of catabolically orthogonal succinogenic specialist strains. Concentrations of **(A)** glucose, **(B)** xylose, **(C)** succinate, and **(D)** OD_550nm_ measured in the fermentation broth for KJglc:KJxyl co-cultures using an initial inoculum ratio of 1:1 (closed circles), 1:50 (closed dashed triangle), 1:100 (asterisk), and 2:1 (open squares). All fermentations were performed in batch mode and in mineral salts media. Data points represent the arithmetic mean of three replicates, and the error bars represent one standard deviation.

KJglc and KJxyl were next combined to develop a synthetic co-culture for producing SA from glucose–xylose mixtures, again employing the same population-level tuning strategy in order to optimize sugar co-utilization. Based on the outcomes revealed for LA production, initial inoculation ratios of 1:1, 1:50, and 1:100 KJglc:KJxyl were first explored. As shown in Figure 5B and Table 2, total xylose utilization and *Q_*X*__*yl*_* were similar for each of the 1:1, 1:50, and 1:100 co-cultures (each ∼87% and ∼0.67 g L^–1^ h^–1^, respectively) and close to that of the KJxyl monoculture. Meanwhile, however, total glucose utilization unexpectedly declined across this initial series of co-cultures (Figure 5A). For instance, compared to 1:1 co-cultures, total glucose utilization dropped by 80 and 94% in the 1:50 and 1:100, respectively; while all three co-cultures displayed reduced maximum *Q_*G*__*l*__*c*_* relative to KJglc monoculture (Figure 5B and Table 2). Overall, total sugar utilization was 91, 39, and 31% for the 1:1, 1:50, and 1:100 co-cultures, respectively (compared to 71% for KJglc monocultures; Table 2), with the highest final SA titers reaching 84 g L^–1^ ± 1 at the 1:1 ratio (at least two-fold greater than by 1:50 or 1:100) along with a maximum *Q*_*SA*_ of 1.3 ± 0.1 g L^–1^ h^–1^ (Figure 5C and Table 2). Interestingly, in contrast to the above LA co-cultures as well as our previous work (Flores et al., 2019), increased initial relative abundance of the xylose specialist did not result in enhanced xylose utilization or, in this case, improved production of SA (Figures 5B,C). This is likely because, in contrast to TGxyl, KJxyl displays much greater fitness, as demonstrated, for example, by its ability to accumulate twice as much biomass during monoculture fermentations [2.7 ± 0.2 gDCW L^–1^ vs. 1.1 ± 0.1 gDCW L^–1^ for TGxyl and 1.7 ± 0.1 gDCW L^–1^ for LYglc1, a previously engineered ethanologenic xylose specialist (Flores et al., 2019)]. To test if it was in fact the relative activity of the glucose specialist that instead limited the overall performance of this SA-producing co-culture, an initial inoculum ratio of 2:1 KJglc:KJxyl was at last explored. While the 2:1 co-culture utilized glucose at a faster rate and consumed 100% of provided glucose by 96 h (compared to 44 h for KJglc monoculture and KJ122), total xylose utilization, on the other hand, dropped to just 46% overall (about half of that consumed by the 1:1 co-culture; Figure 5B and Table 2). Based on this outcome, it was determined that the optimal initial inoculum ratio for this specific co-culture was close to 1:1. Meanwhile, the finding that a unique optimum initial population ratio was required for the developed LA- and SA-producing co-cultures is not altogether surprising and likely reflects the fact that the relative fitness levels differ between the two strains that make up each pair.

For comparison, Xia et al. (2015) previously developed an *E*. *coli* co-culture to convert glucose and xylose to SA via a two-stage, aerobic–anaerobic fed-batch process. Specifically, a mixture composed of ∼30 g L^–1^ glucose and ∼10 g L^–1^ xylose were first utilized aerobically for growth (producing no succinate), before then switching to anaerobic conditions to produce SA (∼15 g L^–1^ glucose and ∼5 g L^–1^ xylose were provided initially and then periodically added over 80 h; Table 3). Total sugar addition to this process over its 115 h duration was ∼100 g L^–1^ (3:1 glucose:xylose by mass), and final SA titers reached ∼45 g L^–1^. From a bioprocessing perspective, while this two-stage approach has proven to be effective, use of a process that can operate simple batch mode, such as the co-culture systems presented here, simplifies operation and control of the process. Similar to LA production, researchers have also engineered generalist strains to produce SA efficiently from sugar mixtures. For example, KJ122 further engineered to enhance conversion of a series of glucose–xylose mixtures (each 100 g L^–1^ total with 1:1, 2:1, or 3:1 glucose:xylose by mass) to up to ∼84 g L^–1^ SA using a combination of genetic engineering and adaptive laboratory evolution (Khunnonkwao et al., 2018). As discussed above, however, the current co-culture strategy is appealing due to its ability to facilitate effective catabolism of sugar mixtures through population tuning.

**FIGURE 6:**
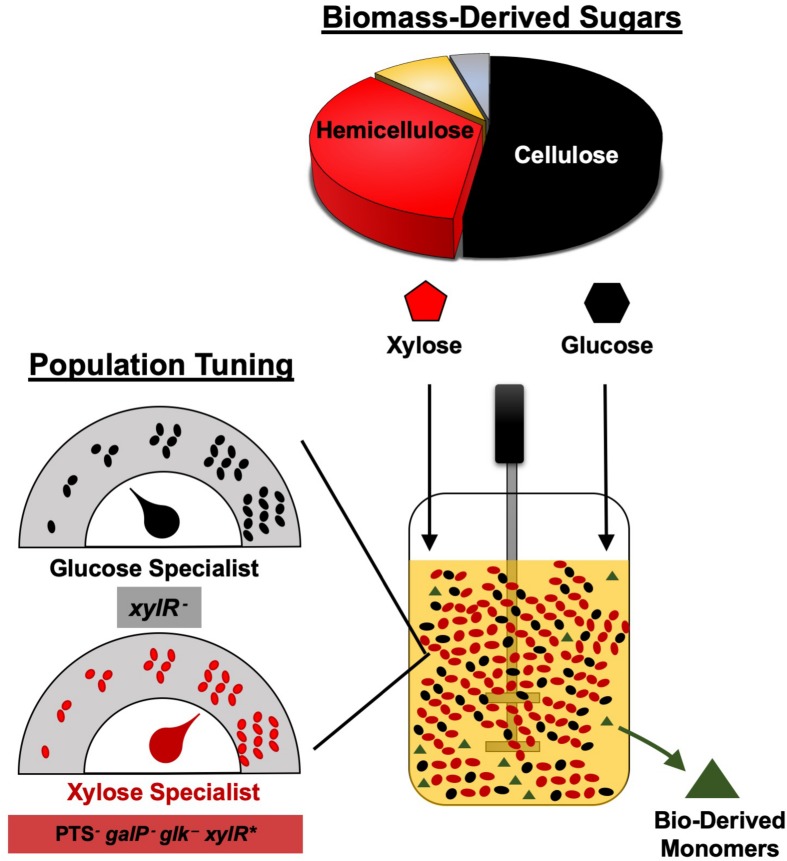
Tuning the initial inoculum ratio enables enhanced bioplastic monomer production from glucose-xylose mixtures by co-cultures of catabolically-orthogonal specialist strains.

Overall, we have demonstrated the broad utility of engineering co-cultures composed of catabolically orthogonal *E*. *coli* strains for efficiently converting sugar mixtures into LA and SA, two important bioplastic monomers. Initial inoculum ratio was revealed to be an important design parameter for maximizing co-culture performance, the optimum value of which is unique to each specialist pair and can vary by even several orders of magnitude depending on relative phenotypic differences between member strains (Figure 6). Ultimately, by applying a population-level tuning strategy to balance rates of glucose and xylose co-utilization, both co-culture systems developed here were capable of fermenting a 100 g L^–1^ glucose–xylose mixture at ∼91% conversion to either LA or SA at high rates and yields. This population-level tuning strategy was simple to implement experimentally and should similarly prove useful in other co-culture applications. Holistically, this work contributes to an improved understanding of the behaviors of synthetic microbial consortia as enhanced bioproduction platforms for renewable fuels and chemicals from non-food carbohydrates. Ultimately, however, the ability to elucidate and understand the nature and potential importance of interstrain interactions and/or metabolite exchanges (Herre et al., 1999; Ponomarova and Patil, 2015; Scott and Hasty, 2016) will likely be important to further optimize these and other co-culture systems.

## Materials and Methods

### Strain Construction

All *E. coli* strains and plasmids used in this study are presented in Table 1. A list of primers used is presented in Supplementary Table S1. The xylose specialist strain (Δ*galP* Δ*ptsI glk:kan^*R*^ xylR*:*xylR*^∗^) derived from KJ122 was initially found to grow poorly in media containing glucose–xylose and was accordingly adapted for improved growth under the conditions of interest. Growth was found to be significantly improved after performing just a single transfer, after which one clone, designated as KJxyl, was isolated. All chromosomal modifications were conducted using one- or two-step integration processes (Datsenko and Wanner, 2000; Sievert et al., 2017). Plasmid pXW001, containing a *cat-sacB* cassette, or strain T-SACK, containing a *tetA-sacB* cassette, were used as the PCR template to generate DNA fragments for primary integration into chromosomal sites of interest (Li et al., 2013; Sievert et al., 2017). Primary integration fragments contained the *cat-sacB* or *tetA-sacB* cassette flanked by 50-bp homology sequences from both upstream and downstream regions of the gene of interest. To eliminate the integrated *cat-sacB* or *tetA-sacB* cassette, providing markerless gene deletions, secondary integration fragments were generated containing 500-bp homology sequences from both upstream and downstream regions of the gene of interest, as generated via fusion PCR. Plasmid pKD46, expressing λ-red recombinase, was used to facilitate all chromosomal integrations via double-crossover recombination, as previously described (Datsenko and Wanner, 2000). During both primary and secondary chromosomal integrations, cultures were inoculated in a 250 ml flask containing 25 ml Luria Broth (LB), 50 g L^–1^ arabinose, and 50 mg L^–1^ ampicillin and incubated at 30°C with shaking at 150 rpm until the optical density at 550 nm (OD_550_) of the cultures reached ~0.5. To prepare competent cells, cultures were subsequently centrifuged (5 min, 6,750 × g, 4°C), the supernatant was discarded, and the remaining cell pellet was resuspended in 20 ml of 4°C water. The described spin-wash cycle was repeated three times. On the last wash, all the supernatant was discarded except ~150–200 μl of the remaining supernatant which was used to resuspend pelleted cells. For electroporation, 40 μl of competent cells were combined with 100–200 ng of DNA. Following electroporation, cells were transferred to a sterile test tube containing 1 ml LB and incubated at 30°C for 4 h. Cells were then plated on LB plates containing the appropriate antibiotic. Colony PCR and Sanger sequencing were used to verify positive clones after selecting for appropriate antibiotic resistance during primary integration and sucrose insensitivity (10% w/v) and loss of antibiotic resistance during secondary integration.

### Cultivation Conditions

Monoculture and co-culture batch fermentations were conducted in a pH (7.0)- and temperature (37°C)-controlled vessel containing 300 ml of modified AM1 mineral salt medium (Martinez et al., 2007; Yomano et al., 2009) containing twice the ammonium phosphate [38.8 mM (NH_4_)_2_H_2_PO_4_ and 15.1 mM (NH_4_)H_2_PO_4_] and 67 g L^–1^ glucose and 33 g L^–1^ xylose (Flores et al., 2019; Martinez et al., 2019). pH was maintained by automatic addition of 6 M KOH for LA-producing cultures and a mixture of 6 M KOH and 3 M K_2_CO_3_ (1:4 ratio by volume) for SA-producing cultures, as previously described (Grabar et al., 2006; Jantama et al., 2008). From –80°C frozen stocks, strains were streaked onto AM1 agar plates supplemented with 100 mM MOPS and 20 g L^–1^ glucose or 20 g L^–1^ xylose. Agar plates were placed inside a sealed canister filled with argon gas and incubated at 37°C for 16–24 h. Seed cultures were grown in AM1 medium containing 100 mM MOPS, 10 g L^–1^ glucose, and 10 g L^–1^ xylose and incubated at 37°C with shaking at 120 rpm for ∼12–16 h. Cells were harvested by centrifugation (5 min, 6,750 × g, 4°C) and resuspended in 300 ml fresh media. All monoculture and co-culture fermentations were seeded using a total initial OD_550mn_ of 0.05 (∼0.022 gDCW L^–1^).

### Analytical Methods

Cell growth was quantified using a UV/Vis spectrophotometer (Beckman Coulter DU-730, Indianapolis, IN, United States). Sugar and product concentrations were determined by high-performance liquid chromatography (HPLC; Thermo Fisher Scientific and UltiMate 3000, Waltham, MA, United States) equipped with a refractive index detector. Analyte separation was performed using an Aminex HPX-87H column (Bio-Rad Laboratories, Hercules, CA, United States) maintained at 45°C and a mobile phase consisting of 5 mM H_2_SO_4_ flowing at a constant rate of 0.4 ml min^–1^. External standards prepared in house were used to quantify substrate and product concentrations. All experiments were performed in at least triplicates, and the average and standard deviation are shown in figures and tables.

## Data Availability Statement

The datasets generated for this study are available on request to the corresponding author.

## Author Contributions

AF, DN, and XW designed the experiments, analyzed the data, and wrote the manuscript. AF constructed all strains and performed all experiments with the assistance of HC, RM, MO, EA, AG, and MM.

## Conflict of Interest

The authors declare that the research was conducted in the absence of any commercial or financial relationships that could be construed as a potential conflict of interest.

## References

[B1] Abdel-Rahman M. A. Tashiro Y. Sonomoto K. (2011). Lactic acid production from lignocellulose-derived sugars using lactic acid bacteria: overview and limits. *J. Biotechnol.* 156 286–301. 10.1016/j.jbiotec.2011.06.017 21729724

[B2] Abdel-Rahman M. A. Tashiro Y. Sonomoto K. (2013). Recent advances in lactic acid production by microbial fermentation processes. *Biotechnol. Adv.* 31 877–902. 10.1016/j.biotechadv.2013.04.002 23624242

[B3] Ahn J. H. Jang Y. S. Lee S. Y. (2016). Production of succinic acid by metabolically engineered microorganisms. *Curr. Opin. Biotechnol.* 42 54–66. 10.1016/j.copbio.2016.02.034 26990278

[B4] Awasthi D. Wang L. Rhee M. S. Wang Q. Chauliac D. Ingram L. O. (2018). Metabolic engineering of *Bacillus subtilis* for production of D-lactic acid. *Biotechnol. Bioeng.* 115 453–463. 10.1002/bit.26472 28986980

[B5] Camacho-Zaragoza J. M. Hernandez-Chavez G. Moreno-Avitia F. Ramirez-Iniguez R. Martinez A. Bolivar F. (2016). Engineering of a microbial coculture of *Escherichia coli* strains for the biosynthesis of resveratrol. *Microb. Cell Fact.* 15:163. 27680538 10.1186/s12934-016-0562-z PMC5041211

[B6] Chappell T. C. Nair N. U. (2017). Co-utilization of hexoses by a microconsortium of sugar-specific *E. coli strains*. *Biotechnol. Bioeng.* 114 2309–2318. 10.1002/bit.26351 28600864

[B7] Datsenko K. A. Wanner B. L. (2000). One-step inactivation of chromosomal genes in *Escherichia coli* K-12 using PCR products. *Proc. Natl. Acad. Sci. U.S.A.* 97 6640–6645. 10.1073/pnas.120163297 10829079 PMC18686

[B8] Eiteman M. A. Lee S. A. Altman E. (2008). A co-fermentation strategy to consume sugar mixtures effectively. *J. Biol. Eng.* 2:3. 10.1186/1754-1611-2-3 18304345 PMC2266900

[B9] Eiteman M. A. Lee S. A. Altman R. Altman E. (2009). A substrate-selective co-fermentation strategy with *Escherichia coli* produces lactate by simultaneously consuming xylose and glucose. *Biotechnol. Bioeng.* 102 822–827. 10.1002/bit.22103 18828178

[B10] Es I. Mousavi Khaneghah A. Barba F. J. Saraiva J. A. Sant’Ana A. S. Hashemi S. M. B. (2018). Recent advancements in lactic acid production - a review. *Food Res. Int.* 107 763–770. 10.1016/j.foodres.2018.01.001 29580545

[B11] Flores A. D. Ayla E. Z. Nielsen D. R. Wang X. (2019). Engineering a synthetic, catabolically orthogonal coculture system for enhanced conversion of lignocellulose-derived sugars to ethanol. *ACS Synth. Biol.* 8 1089–1099. 10.1021/acssynbio.9b00007 30979337

[B12] Grabar T. B. Zhou S. Shanmugam K. T. Yomano L. P. Ingram L. O. (2006). Methylglyoxal bypass identified as source of chiral contamination in l(+) and d(-)-lactate fermentations by recombinant *Escherichia coli*. *Biotechnol. Lett.* 28 1527–1535. 10.1007/s10529-006-9122-7 16868860

[B13] Herre E. A. Knowlton N. Mueller U. G. Rehner S. A. (1999). The evolution of mutualisms: exploring the paths between conflict and cooperation. *Trends Ecol. Evol.* 14 49–53. 10.1016/s0169-5347(98)01529-8 10234251

[B14] Ishida N. Suzuki T. Tokuhiro K. Nagamori E. Onishi T. Saitoh S. (2006). D-lactic acid production by metabolically engineered *Saccharomyces cerevisiae*. *J. Biosci. Bioeng.* 101 172–177. 10.1263/jbb.101.172 16569615

[B15] Jansen M. L. van Gulik W. M. (2014). Towards large scale fermentative production of succinic acid. *Curr. Opin. Biotechnol.* 30 190–197. 10.1016/j.copbio.2014.07.003 25118136

[B16] Jantama K. Zhang X. Moore J. C. Shanmugam K. T. Svoronos S. A. Ingram L. O. (2008). Eliminating side products and increasing succinate yields in engineered strains of *Escherichia coli* C. *Biotechnol. Bioeng.* 101 881–893. 10.1002/bit.22005 18781696

[B17] Jones J. A. Vernacchio V. R. Collins S. M. Shirke A. N. Xiu Y. Englaender J. A. (2017). Complete biosynthesis of anthocyanins using *E. coli Polycultures*. *mBio* 8:e00621-17. 10.1128/mBio.00621-17 28588129 PMC5461408

[B18] Khunnonkwao P. Jantama S. S. Kanchanatawee S. Jantama K. (2018). Re-engineering *Escherichia coli* KJ122 to enhance the utilization of xylose and xylose/glucose mixture for efficient succinate production in mineral salt medium. *Appl. Microbiol. Biotechnol.* 102 127–141. 10.1007/s00253-017-8580-2 29079860

[B19] Li X. T. Thomason L. C. Sawitzke J. A. Costantino N. Court D. L. (2013). Positive and negative selection using the tetA-sacB cassette: recombineering and P1 transduction in *Escherichia coli*. *Nucleic Acids Res.* 41:e204. 10.1093/nar/gkt1075 24203710 PMC3905872

[B20] Litsanov B. Kabus A. Brocker M. Bott M. (2012). Efficient aerobic succinate production from glucose in minimal medium with *Corynebacterium glutamicum*. *Microb. Biotechnol.* 5 116–128. 10.1111/j.1751-7915.2011.00310.x 22018023 PMC3815278

[B21] Lu H. Villada J. C. Lee P. K. H. (2019). Modular metabolic engineering for biobased chemical production. *Trends Biotechnol.* 37 152–166. 10.1016/j.tibtech.2018.07.003 30064888

[B22] Lynd L. R. (2017). The grand challenge of cellulosic biofuels. *Nat. Biotechnol.* 35 912–915. 10.1038/nbt.3976 29019992

[B23] Maleki N. Safari M. Eiteman M. A. (2018). Conversion of glucose-xylose mixtures to pyruvate using a consortium of metabolically engineered *Escherichia coli*. *Eng. Life Sci.* 18 40–47. 10.1002/elsc.201700109 PMC6999249 32624859

[B24] Martinez A. Grabar T. B. Shanmugam K. T. Yomano L. P. York S. W. Ingram L. O. (2007). Low salt medium for lactate and ethanol production by recombinant *Escherichia coli* B. *Biotechnol. Lett.* 29 397–404. 10.1007/s10529-006-9252-y 17160622

[B25] Martinez R. Flores A. D. Dufault M. E. Wang X. (2019). The XylR variant (R121C and P363S) releases arabinose-induced catabolite repression on xylose fermentation and enhances coutilization of lignocellulosic sugar mixtures. *Biotechnol. Bioeng*. 116, 3476–3481. 10.1002/bit.27144 31429933

[B26] Nieves L. M. Panyon L. A. Wang X. (2015). Engineering sugar utilization and microbial tolerance toward lignocellulose conversion. *Front. Bioeng. Biotechnol.* 3:17. 10.3389/fbioe.2015.00017 25741507 PMC4332379

[B27] Okano K. Tanaka T. Ogino C. Fukuda H. Kondo A. (2010). Biotechnological production of enantiomeric pure lactic acid from renewable resources: recent achievements, perspectives, and limits. *Appl. Microbiol. Biotechnol.* 85 413–423. 10.1007/s00253-009-2280-5 19826806

[B28] Ponomarova O. Patil K. R. (2015). Metabolic interactions in microbial communities: untangling the Gordian knot. *Curr. Opin. Microbiol.* 27 37–44. 10.1016/j.mib.2015.06.014 26207681

[B29] Roell G. W. Zha J. Carr R. R. Koffas M. A. Fong S. S. Tang Y. J. (2019). Engineering microbial consortia by division of labor. *Microb. Cell Fact.* 18:35. 10.1186/s12934-019-1083-3 30736778 PMC6368712

[B30] Sawisit A. Jantama K. Zheng H. Yomano L. P. York S. W. Shanmugam K. T. (2015). Mutation in *galP* improved fermentation of mixed sugars to succinate using engineered *Escherichia coli* AS1600a and AM1 mineral salts medium. *Bioresour. Technol.* 193 433–441. 10.1016/j.biortech.2015.06.108 26159300

[B31] Scott S. R. Hasty J. (2016). Quorum sensing communication modules for microbial consortia. *ACS Synth. Biol.* 5 969–977. 10.1021/acssynbio.5b00286 27172092 PMC5603278

[B32] Sievert C. Nieves L. M. Panyon L. A. Loeffler T. Morris C. Cartwright R. A. (2017). Experimental evolution reveals an effective avenue to release catabolite repression via mutations in XylR. *Proc. Natl. Acad. Sci. U.S.A.* 114 7349–7354. 10.1073/pnas.1700345114 28655843 PMC5514714

[B33] Song S. Park C. (1997). Organization and regulation of the D-xylose operons in *Escherichia coli* K-12: XylR acts as a transcriptional activator. *J. Bacteriol.* 179 7025–7032. 10.1128/jb.179.22.7025-7032.1997 9371449 PMC179643

[B34] Utrilla J. Vargas-Tah A. Trujillo-Martinez B. Gosset G. Martinez A. (2016). Production of D-lactate from sugarcane bagasse and corn stover hydrolysates using metabolic engineered *Escherichia coli* strains. *Bioresour. Technol.* 220 208–214. 10.1016/j.biortech.2016.08.067 27573474

[B35] Wang L. York S. W. Ingram L. O. Shanmugam K. T. (2019). Simultaneous fermentation of biomass-derived sugars to ethanol by a co-culture of an engineered *Escherichia coli* and *Saccharomyces cerevisiae*. *Bioresour. Technol.* 273 269–276. 10.1016/j.biortech.2018.11.016 30448678

[B36] Wang Q. Ingram L. O. Shanmugam K. T. (2011). Evolution of D-lactate dehydrogenase activity from glycerol dehydrogenase and its utility for D-lactate production from lignocellulose. *Proc. Natl. Acad. Sci. U.S.A.* 108 18920–18925. 10.1073/pnas.1111085108 22065761 PMC3223474

[B37] Xia T. Altman E. Eiteman M. A. (2015). Succinate production from xylose-glucose mixtures using a consortium of engineered *Escherichia coli*. *Eng. Life Sci.* 15 65–72. 10.1002/elsc.201400113

[B38] Yomano L. P. York S. W. Shanmugam K. T. Ingram L. O. (2009). Deletion of methylglyoxal synthase gene (*mgsA*) increased sugar co-metabolism in ethanol-producing *Escherichia coli*. *Biotechnol. Lett.* 31 1389–1398. 10.1007/s10529-009-0011-8 19458924 PMC2721133

[B39] Zhang H. Pereira B. Li Z. Stephanopoulos G. (2015). Engineering *Escherichia coli* coculture systems for the production of biochemical products. *Proc. Natl. Acad. Sci. U.S.A.* 112 8266–8271. 10.1073/pnas.1506781112 26111796 PMC4500268

[B40] Zhou K. Qiao K. J. Edgar S. Stephanopoulos G. (2015). Distributing a metabolic pathway among a microbial consortium enhances production of natural products. *Nat. Biotechnol.* 33 377–383. 10.1038/nbt.3095 25558867 PMC4867547

