# Supplementary material for: Catabolic Division of Labor Enhances Production of D-Lactate and Succinate From Glucose-Xylose Mixtures in Engineered Escherichia coli Co-culture Systems
Source: Front Bioeng Biotechnol. 2020 May 5;8:329. doi: 10.3389/fbioe.2020.00329 (PMC7214542; doi:10.3389/fbioe.2020.00329)
Supplement: Supplementary file 1 [file Table_1.docx]

**Catabolic Division of Labor Enhances Production of D-Lactate and Succinate from Glucose-Xylose Mixtures in Engineered *Escherichia coli* Coculture Systems**

**Supplementary Data**


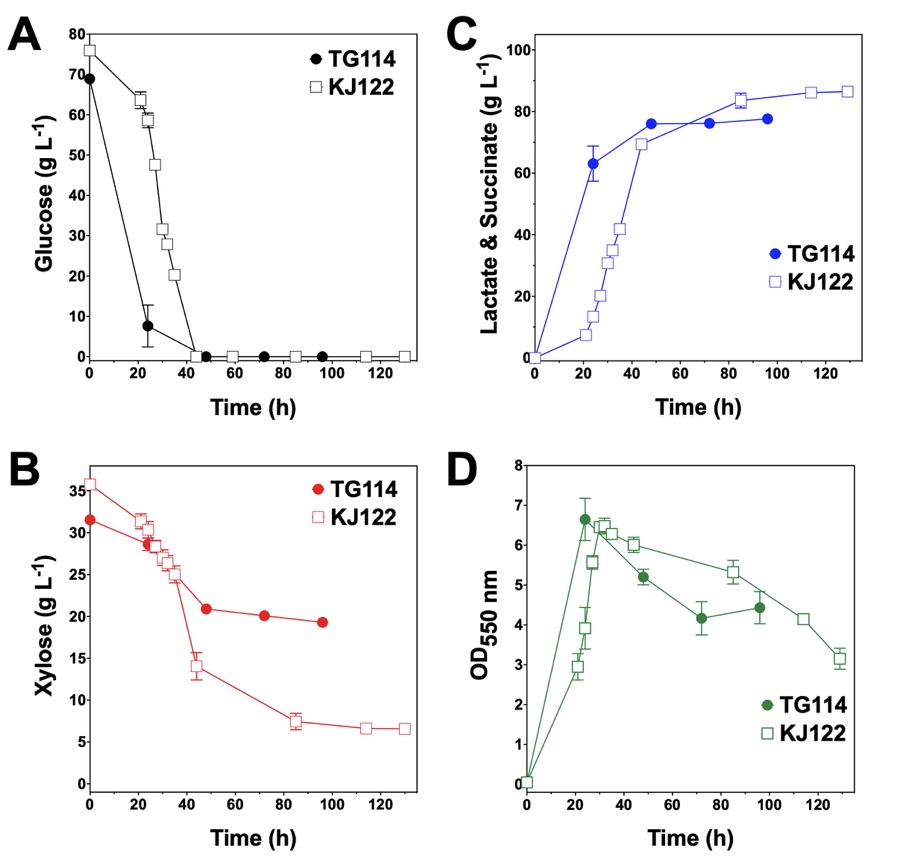


**Figure S1. Fermentation of glucose-xylose mixtures by lactic acid-producing *E*. coli TG144 and succinogenic *E*. *coli* KJ122.** Concentrations of (A) glucose, (B) xylose, (C) succinate and (D) OD_550nm_ measured in the fermentation broth for TG114 (closed circles), KJ122 (open squares). All fermentations were performed in batch mode and in mineral salts media. Data points represent the arithmetic mean of three replicates and error bars represent one standard deviation

**
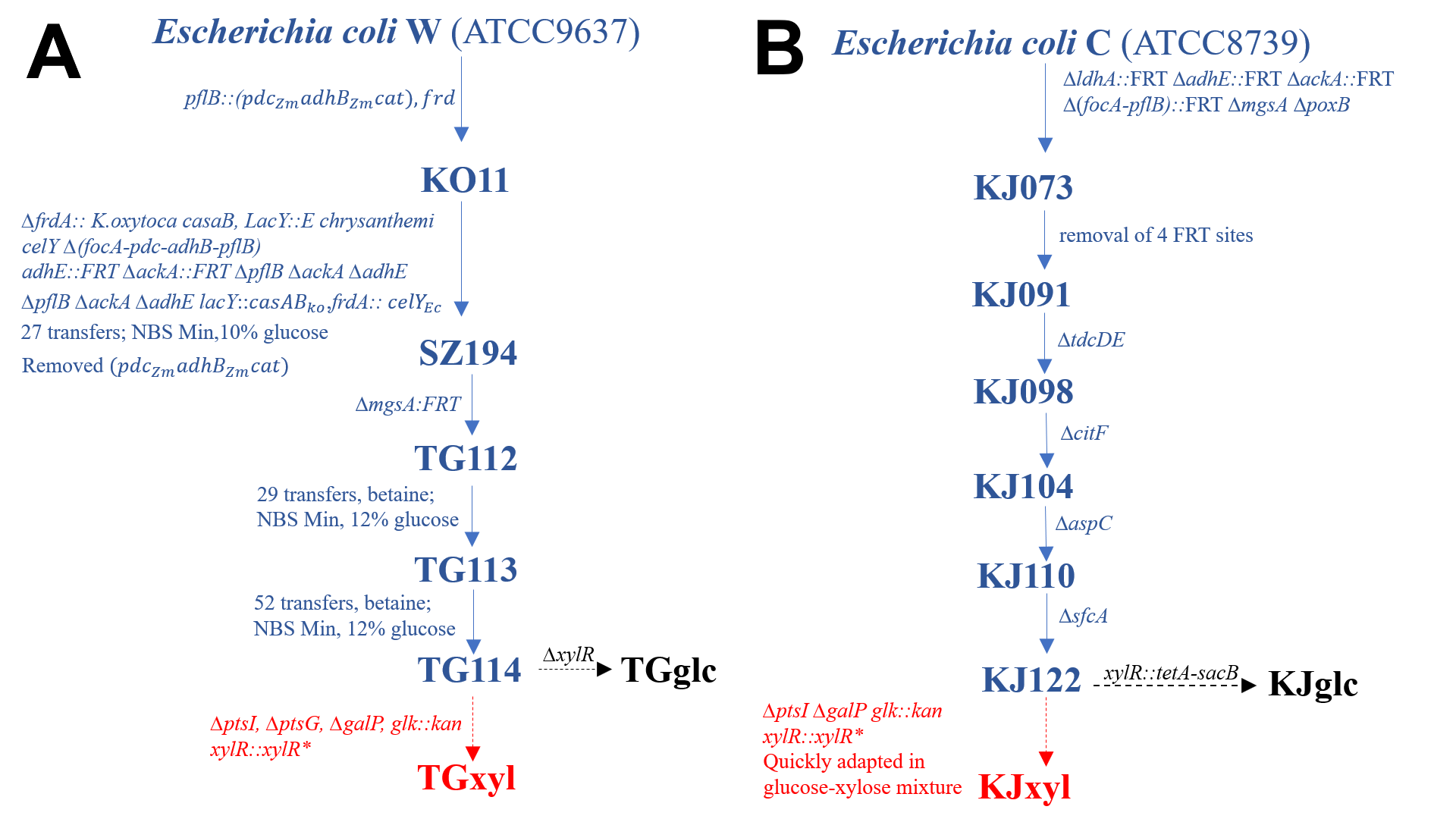
Figure S2. Strain lineage for the specialists developed and used in this study.** (A) Strain lineage for D-lactate producing sugar specialists. (B) Strain lineage for succinate producing sugar specialists. Previously engineered strains are indicated by blue font. And solid arrows Glucose specialists are indicated by black font and dashed arrows. Xylose specialists are indicated by red font and dashed arrows.

**Table S1. List of primers used in this study.**

| ***Primer Name*** | **Primer Sequence** |
| --- | --- |
| ***ptsI* deletion** |  |
| *cat-sacB* F | GAGTAATTTCCCGGGTTCTTTTAAAAATCAGTCACAAGTAAGGTAGGGTTTCGAGTGTGACGGAAGATCA |
| *cat-sacB* R | ACAAACCCATGATCTTCTCCTAAGCAGTAAATTGGGCCGCATCTCGTGGATTAGCCATTTGCCTGCTTTT |
| *ptsI H1*F | AATCAGTCACAAGTAAGGTAGGGTTTCCACGAGATGCGGCCCAAT T |
| *ptsI H1 R* | CCGGAGTCAGGGTAGACTTG |
| *ptsI H2 F* | AATTGGGCCGCATCTCGTGGAAACCCTACCTTACTTGTGACTGAT |
| *ptsI H2 R* | ACTGTATTGCGCTCTTCGTG |
| ***ptsG* deletion** |  |
| *cat-sacB* F | CACGCGTGAGAACGTAAAAAAAGCACCCATACTCAGGAGCACTCTCAATTTCGAGTGTGACGGAAGATCA |
| *cat-sacB* R | GTAAAAAAGGCAGCCATCTGGCTGCCTTAGTCTCCCCAACGTCTTACGGATTAGCCATTTGCCTGCTTTT |
| *ptsG H1*F | CGGTTACTGGTGGAAACTGACTCAC |
| *ptsG H1 R* | CTTAGTCTCCCCAACGTCTTACGGAAATTGAGAGTGCTCCTGAGTATGGGT |
| *ptsG H2 F* | ACCCATACTCAGGAGCACTCTCAATTTCCGTAAGACGTTGGGGAGACTAAG |
| *ptsG H2 R* | GACAGTCAGTAAAGGGGTGGAATTTGAAC |
| ***galP* deletion** |  |
| *cat-sacB* F | TACTCACCTATCTTAATTCACAATAAAAAATAACCATATTGGAGGGCATCTCGAGTGTGACGGAAGATCA |
| *cat-sacB* R | GATGACTGCAAGAGGTGGCTTCCTCCGCGATGGGAGGAAGCTTGGGGAGATTAGCCATTTGCCTGCTTTT |
| *galP H1*F | GGTCGTGAACATTTCCCGTGG |
| *galP H1 R* | TGGGAGGAAGCTTGGGGAGAGATGCCCTCCAATATGGTTATTTTTTATTGTGAAT |
| *galP H2 F* | ATTCACAATAAAAAATAACCATATTGGAGGGCATCTCTCCCCAAGCTTCCTCCCA |
| *galP H2 R* | CGGTAAGCTGATGCTCCTGG |
| ***xylR* deletion & replacement** |  |
| *cat-sacB* F | TCTCAAAGCCGGTTACGTATTACCGGTTTTGAGTTTTTGCATGATTCAGCTCGAGTGTGACGGAAGATCA |
| *cat-sacB* R | GATAAGGCTTTTGCTCGCATCAGGTGGCTGTGCTGAGTTCCCTGATGTGACCTTAGCCATTTGCCTGCT |
| *tetA-sacB F* | TTTGAGTTTTTGCATGATTCAGCAGGAAAAGAACCTCCTAATTTTTGTTGACACTCTATC |
| *tetA-sacB R* | GTTACCTGATGTGACGCCGACAATTCTCATCATCGATCAAAGGGAAAACTGTCCATATGC |
| *xylR H1*F | CTGTTACTCGGCGGAATGTT |
| *xylR H1 R* | GACGCCGACAATTCTCATCATCGGGTTCCTTTTCCTGCTGAATCATGC |
| *xylR H2 F* | GCATGATTCAGCAGGAAAAGAACCCGATGATGAGAATTGTCGGCGTC |
| *xylR H2 R* | CTTGCTTGAACGCGTAGACA |
| ***lacZ replacement*** |  |
| *cat-sacB* F | TCACACAGGAAACAGCTATGACCATGATTACGGATTCACTGGCCGTCGTTTCGAGTGTGACGGAAGATCA |
| *cat-sacB* R | CATTCGCCATTCAGGCTGCGCAACTGTTGGGAAGGGCGATCGGTGCGGGCCCTTAGCCATTTGCCTGCT |
| ***glk replacement*** |  |
| kan F | ATTTACAGTGTGAGAAAGAATTATTTTGACTTTAGCGGAGCAGTTGAAGAGTGTAGGCTGGAGCTGCTTC |
| kan R | TGATTTAAAAGATTATCGGGAGAGTTACCTCCCGATATAACAGGAAGGATCATATGAATATCCTCCTTAGT |

F, forward primer; R, reverse primer; H1 corresponds to 500 bp upstream of the gene of interests; H2 corresponds to 500 bp downstream of the gene of interests.
